# 

**Published:** 1908-03

**Authors:** 


### THE BRISTOL Medico-Chirurgical Journal.

A JOURNAL OF THE MEDICAL SCIENCES FOR THE WEST OF ENGLAND AND SOUTH WALES

*PUBLISHED UNDER THE AUSPICES OF*

*THE BRISTOL MEDICO-CHIRURGICAL SOCIETY.*

EDITOR:
R. SHINGLETON SMITH, M.D., B.Sc.,
WITH WHOM ARE ASSOCIATED
J. MICHELL CLARKE, M.A., M.D., J. LACY FIRTH, M.S., M.D.,
AND JAMES SWAIN, M.S., M.D.
ASSISTANT-EDITOR:
P. WATSON WILLIAMS, M.D.
EDITORIAL SECRETARY:
JAMES TAYLOR.

"Scire est nescire, nisi id me

Scire alius sciret."

*VOL. XXVI.*

LIBRARY OF CONGRESS

JAN 14 1909

SMITHSONIAN DEPOSIT.

BRISTOL: J. W. ARROWSMITH.
LONDON: J. & A. CHURCHILL, 7 GREAT MARLBOROUGH STREET.
1908.

R31
B8

J.W. Arrowsmith

Publisher

Bristol

### CONTENTS OF VOLUME XXVI.

Page

The Long Fox Lecture: Suppurative Disease in the Nose and Ear. By PATRICK WATSON WILLIAMS, M.D.Lond. .. .. .. 1

Typhoid Fever and Typhoid Carriers. By D. S. DAVIES, M.D.Lond., and I. WALKER HALL, M.D.Vict. .. .. .. .. 39

Operative Interference in Carcinoma of the Pancreas. By JAMES SWAIN, M.S., M.D.Lond., F.R.C.S. .. .. .. 45

The Treatment of Spreading Peritonitis by Drainage, the Fowler Position, and Rectal Instillation of Saline Solution, with Notes of a Second Successful Case. By C. HAMILTON WHITEFORD .. 53

Case of Rupture of Middle Meningeal Artery: Operation; Recovery. By G. MUNRO SMITH .. .. .. .. .. .. 58

Abdominal Fibroma Simulating Splenomegaly. By DAVID A. ALEXANDER, M.B.Edin. .. .. .. .. 60

Is Opsonic Treatment Useful in Phthisis? By JOHN M. H. MUNRO, D.Sc.Lond. .. .. .. .. 97

Calmette's Ophthalmic Reaction in Tuberculosis. By J. M. FORTESCUE-BRICKDALE, M.A., M.D.Oxon. .. .. 112

Some Notes on the Treatment of Hæmoptysis in Pulmonary Tuberculosis. By NEWMAN NEILD, M.B.Vict., M.R.C.P. .. .. 124

Some Clinical Points in the Early Diagnosis of Pulmonary Tuberculosis. By C. MUTHU, M.D. .. .. .. .. .. 131

Case of Filariasis with Abscess. By A. L. FLEMMING .. .. 139

Lachrymal Obstruction: its Results, Dangers and Treatment. By CHARLES GOULDEN, M.A., M.B., M.C.Cantab., F.R.C.S.Eng. 143

Early History of the Bristol Royal Infirmary. By J. PAUL BUSH, C.M.G. .. .. .. .. .. .. 193

A Study of Ten Cases of Operation for Perforated Gastric Ulcer. By CHARLES A. MORTON, F.R.C.S. .. .. .. .. 207

Surgical Aid in Chronic Ulcer of the Stomach. By J. A. NIXON, M.B.Cantab., M.R.C.P.Lond... .. .. 217

CONTENTS.

Page

The Aural Complications of Influenza and their Treatment. By HAROLD F. MOLE, F.R.C.S. .. .. .. .. .. 226

On the Renal Changes in a Case of Hemorrhage into the Pons with Consequent High Blood Pressure. By J. MICHELL CLARKE, M.A., M.D.Cantab., F.R.C.P. .. 230

A Case of Severe Trigeminal Neuralgia Successfully Treated by Excision of the Gasserian Ganglion. By ERNEST W. HEY GROVES, M.S.Lond., F.R.C.S. .. .. 234

Some Complications of Ruptured Liver. By LIONEL SHINGLETON SMITH, M.B.Cantab. .. .. .. 238

Recent Work on Leukæmia and some Allied Affections. By J. MICHELL CLARKE, M.A., M.D.Cantab., F.R.C.P. .. 289

The Causation of Certain Mid-Diastolic Murmurs. By CAREY COOMBS, M.D.Lond., M.R.C.P. .. .. .. 312

Notes on Cases. By T. CARWARDINE, M.S.Lond., F.R.C.S. .. 317

Dilatation of Posterior Cerebral Vessels with Basal Hemorrhage in a Girl aged Sixteen. By T. M. CARTER, M.D. .. .. .. 322

A Medical Naval Volunteer on Duty at Quebec, July, 1908. By W. KENNETH WILLS, M.B., B.C.Cantab. .. .. .. 327

PROGRESS OF THE MEDICAL SCIENCES .. 61, 152, 241, 335

REVIEWS OF BOOKS .. .. .. .. 69, 164, 253, 343

EDITORIAL NOTES .. .. .. .. 83, 173, 279, 357

NOTES ON PREPARATIONS FOR THE SICK .. 86, 178, 284, 364

THE LIBRARY OF THE BRISTOL MEDICO-CHIRURGICAL SOCIETY .. .. .. .. 89, 183, 286, 367

MEETINGS OF SOCIETIES .. .. .. .. 92, 186, 369

OBITUARY NOTICES .. .. .. .. .. .. .. 376

LOCAL MEDICAL NOTES .. .. .. .. 94, 190, 288, 378

### The Library of the Bristol Medico-Chirurgical Society.

---

*The following donations have been received since the publication of the List in December:*

*February 29th,* 1908.

| | |
|---|---|
| J. Michell Clarke, M.D. (1) .. .. .. .. | 1 volume. |
| H. Griffiths (2) .. .. .. .. .. .. | 1 ,, |
| L. M. Griffiths .. .. .. .. .. .. | 1 ,, |
| The Editor of *Albany Medical Annals* (3) .. .. | 1 ,, |
| The Medical Officer of the London County Council (4) | 1 ,, |
| R. Shingleton Smith, M.D. (5) .. .. .. | 1 ,, |
| James Willing, Jun., Ltd. (6) .. .. .. | 1 ,, |

### SIXTY-SEVENTH LIST OF BOOKS.

The titles of books mentioned in previous lists are not repeated.

The figures in brackets refer to the figures after the names of the donors and show by whom the volumes are presented. The books to which no such figures are attached have either been bought from the Library Fund, or received through the *Journal.*

| | | |
|---|---|---|
| **Allen, R. W.** | .. *The Opsonic Method of Treatment* .. .. | 1907 |
| **Barker, L. F.** | .. *Anatomical Terminology* [B.N.A.] .. .. | 1907 |

**Barton, G. A. H.** *Guide to Administration of Ethyl Chloride* 2nd Ed. 1907
**Bramwell, B.** .. .. *Clinical Studies* .. .. .. .. (5) Vol. V. 1907
**Brockbank, E. M.** *Life Insurance and General Practice* .. .. 1908
**Calmette, A.** .. .. *Les Venins* .. .. .. .. .. .. .. .. .. .. 1907
**Clarke, J. J.** .. .. *Protozoa and Disease* .. .. .. .. Pt. II. 1908
**Cunning, J.** .. .. *Aids to Surgery* .. .. .. .. .. 2nd Ed. 1908
**Fagge, C. H.** .. *The Pocket Anatomy* .. .. .. .. 6th Ed. 1908
**Fortescue-Brickdale, F. Francis and J. M.** *The Chemical Basis of Pharmacology* .. .. .. .. .. .. .. .. 1908
**Francis and J. M. Fortescue-Brickdale, F.** *The Chemical Basis of Pharmacology* .. .. .. .. .. .. .. .. 1908
**Griffiths, H.** .. .. *Plenum Ventilation* .. .. .. .. .. (2) [1905]
**Hood, D. W. C.** .. *Some of the Clinical Aspects of Pneumonia* .. 1907
**Jacobson and R. P. Rowlands, W. H. A.** *The Operations of Surgery.* 2 vols., 5th Ed. 1907
**Kehr, H.** .. .. *Drei Jahre Gallensteinchirurgie* .. .. .. 1908
**Laveran, A.** .. .. *Traité du Paludisme* .. .. .. 2me Éd. 1907
**Laveran and F. Mesnil, A.** *Trypanosomes and Trypanosomiasis* (Tr. by D. Nabarro) .. .. .. .. .. .. 1907
**McCullock, H. D.** .. *Stray Leaves, and some Fruit on Cancer and Tuberculosis* .. .. .. .. .. .. .. .. .. 1907
**Macfie, R. C.** .. .. *The Romance of Medicine* .. .. .. .. .. 1907
**Mesnil, A. Laveran and F.** *Trypanosomes and Trypanosomiasis.* (Tr. by D. Nabarro) .. .. .. .. .. 1907
**Metchnikoff, É.** .. *Prolongation of Life.* (Ed. by P. C. Mitchell) 1907
**Meyer und H. Rieder, E.** *Atlas der klinischen Mikroskopie des Blutes* .. .. .. .. .. Zweite Aufl. 1907
**Oliver, G.** .. .. *Studies in Blood Pressure* .. .. 2nd Ed. 1908
**Richmond, G. E.** .. *Disease, its Cause and Prevention* .. .. 1907
**Rieder, E. Meyer und H.** *Atlas der klinischen Mikroskopie des Blutes* .. .. .. .. .. Zweite Aufl. 1907
**Rogers, L.** .. .. *Fevers in the Tropics* .. .. .. .. .. .. .. 1908
**Rowlands, W. H. A. Jacobson and R. P.** *The Operations of Surgery.* 2 vols., 5th Ed. 1907
**Sherrington, C. S.** *The Integrative Action of the Nervous System* 1906
**Tilley, H.** .. .. *Diseases of the Nose and Throat* .. .. .. 1908
**Tunstall, F. J. Warwick and A. C.** *First Aid* .. .. .. .. 5th Ed. 1908
**Tweedy and G. T. Wrench, E. H.** *Rotunda Practical Midwifery* .. 1908
**Vierordt, H.** .. .. *Daten und Tabellen fur Mediziner* .. .. Dritte Aufl. 1906
**Wallis, C. E.** .. *The Care of the Teeth in Schools* .. .. .. 1908
**Warwick and A. C. Tunstall, F. J.** *First Aid.* .. .. .. 5th Ed. 1908
**Wrench, E. H. Tweedy and G. T.** *Rotunda Practical Midwifery* .. 1908
**Wyllie, J.** .. .. *Tumours of the Cerebellum* .. .. .. .. .. 1908

### TRANSACTIONS, REPORTS, JOURNALS, &c.

| | | |
|---|---|---|
| Albany Medical Annals | (3) Vol. XXVIII. | 1907 |
| American Association of Obstetricians and Gynecologists, Transactions of the | Vol. XIX. | 1907 |
| American Laryngological Association, Transactions of the | | 1907 |
| American Ophthalmological Society, Transactions of the | Vol. XI. Pt. 2 | 1907 |
| American Surgical Association, Transactions of the | Vol. XXV. | 1907 |
| Association of American Physicians, Transactions of the | Vol. XXII. | 1907 |
| Boston Medical and Surgical Journal, The | Vol. CLVII. | 1907 |
| Bristol Directory, Wright's | | 1908 |
| British Almanac, The | | 1908 |
| British Gynæcological Journal | Supplement to Vol. XXII. | 1907 |
| British Medical Journal, The | Vol. II. for | 1907 |
| Clinical Society of London, Transactions of the | Vol. XL. | 1907 |
| Edinburgh Medical Journal, The | N.S., Vol. XXII. | 1907 |
| Gazette hebdomadaire des Sciences médicales de Bordeaux | Tom. XXVIII. | 1907 |
| Glasgow Medical Journal, The | Vol. LXVIII. | 1907 |
| Hazell's Annual | | 1908 |
| Hospitals— | | |
| Guy's Hospital Reports | Vol. LXI. | 1907 |
| Johns Hopkins Hospital Bulletin, The | Vol. XVIII. | 1907 |
| Journal of Anatomy and Physiology, The | (1) Vol. XXVI. | 1892 |
| Journal of Experimental Medicine, The | Vol. IX. | 1907 |
| Lancet, The | Vol. II. for | 1907 |
| Library, The | Vol. VIII. | 1907 |
| London County Council, Annual Report of M.O.H. for 1906 | (4) | 1907 |
| London Medical Journal, West | Vol. XII. | 1907 |
| Medical Directory, The | | 1908 |
| Medical Library and Historical Journal | Vol. IV. | 1906 |
| Medical Press and Circular, The | Vol. CXXXV. | 1907 |
| Medical Record | Vol. LXXII. | 1907 |
| Medical Review, The | Vol. X. | 1907 |
| Münchener medizinische Wochenschrift | Bd. II. für | 1907 |
| Ophthalmological Transactions | Vol. XXVII. | 1907 |
| Otological Society, Transactions of the | Vol. VIII. | 1907 |
| Practitioner, The | Vol. LXXIX. | 1907 |
| Progressive Medicine | Vol. IV. | 1907 |
| Revue hebdomadaire de Laryngologie | Tom. XXVII. | 1907 |
| Scottish Medical and Surgical Journal, The | Vol. XXI. | 1907 |
| Smithsonian Report for 1906 | | 1907 |
| West London Medical Journal | Vol. XII. | 1907 |
| Whitaker's Almanack | | 1908 |
| Willing's Press Guide | (6) | 1908 |
| Year-Book of the Scientific and Learned Societies, The | | 1907 |

### Local Medical Notes.

**University College, Bristol.**—Recent Examination Results —

M.B. OXFORD.—*Pathology:* B. A. W. Stone.

CONJOINT BOARD.—*Biology:* W. Worger, A. G. Morris. *Pharmacy:* S. Marle, G. H. Piercy, P. S. Tomlinson. *Medicine:* R. G. Vaughan. *Midwifery:* F. S. Scott, H. H. Templeton, S. H. Kingston.

L.D.S.—*Chemistry:* S. Saxton.

Additional Demonstrators of Anatomy: W. H. A. Elliott, M.B., B.S.; C. B. Goulden, M.A., M.B., B.C. Cantab., F.R.C.S. Eng.

Dr. Fortescue-Brickdale has been appointed Director of the Public Health Laboratory of University College, Bristol.

UNIVERSITY COLLEGE UNION.—The various societies and clubs of the College have recently amalgamated under the above name. An excellent athletic ground within reasonable distance of the College has been secured, and it is hoped will be ready for use shortly. The secretary of the Union will be pleased to give any further information regarding membership to any old students of the College who may be interested.

MEDICAL RESEARCH CLUB.—The members of the Medical Research Club invited the medical practitioners of the district to meet Sir Almroth Wright on Friday, February 14th. After the usual club dinner, Sir Almroth addressed a representative audience upon the subject of "Vaccine Therapy." He pointed out that when antiseptics are applied to infected areas the antiseptic enters into combination with the tissues more easily than with the micro-organisms. Hence bacteria often flourish under antiseptic applications. The surgeon's knife was also cited as an ineffectual means of freeing the tissues from microbes; the production of active or passive hyperæmia and similar measures was only of limited utility. The chief aim must be to increase the natural resistance to infection by the injection of dead cultures of organisms isolated from the tissues, and by frequent determinations of the resisting power of the blood. The latter are of importance in the treatment of tuberculosis, and of conditions associated with the less common bacteria, because the individual reaction to vaccines varies considerably, and much harm can be done by empirical dosage. With staphylococcal infections, however, routine injections are permissible. Commencing with a dose of 50—100 millions, another injection of 100—250 millions may be made three days later. The patient generally feels better within twenty-four hours; the pain disappears, and the boil recedes. The dose is in such cases to be regulated by the clinical signs.

After some remarks on the necessity for preventing autoinoculation by preventing movements of the affected part during the treatment, Sir Almroth discussed the relation of the blood stream to the infected foci. It was imperative, he said, that the free, or incised, surfaces of any infected area should be frequently flushed with lymph, and with lymph possessing active bactericidal properties. For this purpose he recommended the internal use of citric acid, and the application of salt and sodium citrate to the external surface (sodium citrate 2 grains, sodium chloride 20 grains, boiled water 1 oz.), the former to lessen the coagulability of the blood, and the latter to promote osmosis.

Dealing next with mixed infections, the frequent association of streptococcus and staphylococcus with tubercle bacilli was instanced as an example of the need for double vaccines in some cases. In lupus, particularly, is it necessary to isolate the associated organisms. Here tuberculin frequently fails to procure resolution of the lesion until a coccal vaccine is added.

**Bristol Royal Infirmary.**—The following recent appointments have been made:—J. E. Shaw, M.B., has been appointed Hon. Consulting Physician. *Resident Obstetric Officer:* A. J. Wright, M.B., B.S. Lond. *Junior House Surgeon:* C. E. K. Herapath, M.R.C.S., L.R.C.P. *Casualty Officer:* C. G. Kemp, M.B., B.S. Durh., M.R.C.S., L.R.C.P.

**Bristol Dispensary.**—E. T. Glenny, M.B., B.S. Lond., L. N. Morris, M.R.C.S., J. H. Pinniger, M.B., B.S. Lond., F. G. Bergin, M.R.C.S., have been appointed Medical Officers to the institution.

**Newport and Monmouthshire Hospital.**—V. A. Crinks, M.R.C.S., L.R.C.P., has been appointed Honorary Surgeon.

**Royal Territorial Medical Service.**—There was a well-attended meeting of the profession held in the Medical Library on Friday, January 10th, to hear Colonel Russell, D.A.D.G. explain the medical aspect of Mr. Haldane's new scheme for the Territorial Forces. He traced in a comprehensive lecture the whole arrangements made for the disposition of the medical corps in war from the home hospitals, across the seas, through the base hospitals, lines of communication, the field hospitals and collecting stations up to the front, and elucidated his speech by plans and maps, and having done so, called especial attention to what was to be done in England from the end of March this year, when the Territorial Medical Service was to be equipped and made ready to take the place of the R.A.M.C., should the whole of the R.A.M.C. or a large portion of it be required for service abroad in case of war. The arrangements briefly are as follows. England, Scotland and Wales are divided into ten areas—"grouped regimental districts"—and each has to provide certain units—cavalry, artillery, engineers, infantry, army service corps and medical service. The area to which Bristol belongs is the 7th Regimental District, and it comprises the counties of Gloucestershire, Worcester,

Warwick, Oxford, Berks and Buckinghamshire. This area has to raise three field ambulances, one cavalry ambulance, and two general hospitals, and Bristol is asked to supply one of the field ambulances and one general hospital. There ought to be no great difficulty in this. The general hospital—only for use in time of war in England, *i.e.* on invasion—will necessitate a permanent staff of three officers—an administrator, a registrar and a quartermaster, and forty-three men, chiefly non-commissioned officers. The rest of the staff will consist of civil hospital physicians and surgeons and specialists, who will in time of war in England take on duties at the general hospital, which will be erected and equipped directly it is wanted. The Medical Faculty of University College was naturally the best committee to nominate the members of this medical and surgical staff, and names in the order of seniority for rank have been sent in by the Medical Faculty for the approval of the Director-General. The rates of remuneration are those of the regular army.

As regards the field ambulance, Colonel Russell addressed a public meeting at the Drill Hall on the following evening, and advocated the formation of such a unit in Bristol. The total number wanted is 250 of all ranks—ten officers, all mounted, one of whom, the quartermaster, need not be a medical man, and about sixteen sergeants and staff-sergeants. The great majority of the F.A. will belong to the bearer division, but there will be a large transport to be raised, and men accustomed to horses are wanted for this branch. It is hoped that by the end of March, when the Counties Association will be ready to take on and maintain the unit, a sufficient number of men will have promised to join to make its recognition by the War Office certain. We have reason to think there will be no difficulty about the officers willing to take a commission ; but should any of our readers who take a keen interest in the work like to join, we recommend them to apply to the authorities without delay. Bristol has heretofore had a very good record in being able to raise volunteer corps of all arms, and we hope that now, when the new body of men that will be so intimately associated with the medical profession is being recruited, we shall find Bristol will not allow her character for energy and loyalty to be called in question. It would be of great assistance if those members of the medical world who can influence the right kind of young men to come forward and join in this good work would not forget to recommend them—for the benefit of themselves, their country, and their King—to present themselves on any evening at the Rifle Drill Hall, and promise to enlist before the end of March. We hope to publish in our next number the names and ranks of those medical officers whose names have been submitted to the Director-General to commission, both on the general hospital staff and in the field ambulance.

### Bristol Medico-Chirurgical Society.

### President.

H. WALDO, M.D., C.M. Aberd.

### President-Elect.

J. MICHELL CLARKE, M.D., M.A. Cantab.

### Honorary Secretary.

J. A. NIXON, M.B., B.A. Cantab.

### Committee.

*Past Presidents.*

L. M. GRIFFITHS.
R. SHINGLETON SMITH, M.D., B.Sc. Lond.
NELSON C. DOBSON.
F. R. CROSS, M.B. Lond.
A. J. HARRISON, M.B. Lond.
ARTHUR W. PRICHARD.
J. E. SHAW, M.B., C.M. Ed.
R. ROXBURGH, M.B. Ed.
W. H. HARSANT.
D. S. DAVIES, M.D. Lond.
BARCLAY J. BARON, M.B., C.M. Edin.
G. MUNRO SMITH.
J. PAUL BUSH, C.M.G.
REGINALD EAGER, M.D. Lond.
JOHN DACRE.
JAMES TAYLOR.

B. M. H. ROGERS, M.D., B.Ch., B.A. Oxon.
P. WATSON WILLIAMS, M.D. Lond.
C. K. RUDGE.
JAMES SWAIN, M.D., M.S. Lond.
G. PARKER, M.D., M.A. Cantab.
I. WALKER HALL, M.D. Vict.

Medical Practitioners wishing to join the Society are requested to communicate with the Hon. Secretary, Dr. NIXON, 18 West Mall, Clifton, Bristol. The Annual Subscription is 21/-.

---

*Extracts from Journal By-laws.*

4.—The *Journal* shall be delivered free to Subscribers and also to Members of the Society whose subscriptions are not in arrear.

6.—The Annual Subscription to the *Journal* for those who pay before the 1st of July shall be 5/-, post free, or 6/- if paid after that date.

---

Communications intended for insertion in the *Journal* should be sent to the Editor, Dr. SHINGLETON SMITH, Deepholm, Clifton Park, Clifton, Bristol.

Books for Review and Exchange Journals should be sent to the Assistant-Editor, Dr. P. WATSON WILLIAMS, Medical Department, University College, Bristol.

Communications referring to the delivery of the *Journal* should be sent to the Editorial Secretary, Mr. JAMES TAYLOR, 36 Alma Road, Clifton, Bristol, to whom Subscriptions are to be paid *in advance*.

Cases for Binding Vols. I.–XXV. are now ready, and may be obtained, price 7*d*. each (postage 2*d*. extra), upon application to J. W. ARROWSMITH, Quay Street, Bristol; or the *Journal* will be bound, including Case, for 1/3 per volume.

### Bristol Medico-Chirurgical Society.

---

### PAST PRESIDENTS.

1874—75 FREDERICK BRITTAN, M.D., B.A. Dub.
1875—76. ROBERT WILLIAM COE.
1876—77. SAMUEL MARTYN, M.D. Ed.
1877—78. AUGUSTIN PRICHARD, M.D. Berlin.
1878—79. HENRY EDWARD FRIPP, M.D. Würzburg.
1879—80. WILLIAM MICHELL CLARKE.
1880—81. JOSEPH GRIFFITHS SWAYNE, M.D. Lond.
1881—82. EDWARD LONG FOX, M.D. Oxon.
1882—83. JAMES GEORGE DAVEY, M.D. St. And.
1883—84. WILLIAM JOHNSTONE FYFFE, M.D., B.A. Dub.
1884—85. GEORGE FORSTER BURDER, M.D. Aberd.
1885—86. WILLIAM HENRY SPENCER, M.D., M.A. Cantab.
1886—87. FRANCIS POOLE LANSDOWN.
1887—88. LEMUEL MATTHEWS GRIFFITHS.
1888—89. ROBERT SHINGLETON SMITH, M.D., B.Sc. Lond.
1889—90. NELSON CONGREVE DOBSON.
1890—91. SAMUEL HENRY SWAYNE.
1891—92. FRANCIS RICHARDSON CROSS, M.B. Lond.
1892—93. EDWARD MARKHAM SKERRITT, M.D., B.S., B.A. Lond.
1893—94. JAMES GREIG SMITH, M.B., C.M., M.A. Aberd., F.R.S.E.
1894—95. ALFRED JAMES HARRISON, M.B. Lond.
1895—96. ARTHUR WILLIAM PRICHARD.
1896—97. ALFRED EDWARD AUST LAWRENCE, M.D., C.M. Aberd.
1897—98. JOHN EDWARD SHAW, M.B., C.M. Ed.
1898—99. ROBERT ROXBURGH, M.B. Ed.
1899—00. WILLIAM HENRY HARSANT.
1900—01. DAVID SAMUEL DAVIES, M.D. Lond.
1901—02. BARCLAY JOSIAH BARON, M.B., C.M. Ed.
1902—03. GEORGE MUNRO SMITH.
1903—04. JAMES PAUL BUSH, C.M.G.
1904—05. REGINALD EAGER, M.D. Lond.
1905—06. JOHN DACRE.
1906—07. JAMES TAYLOR.

1908.

### LIST OF SUBSCRIBERS

TO THE

### Bristol Medico-Chirurgical Journal.

*Those marked * are Members of the Society.*

| | |
|---|---|
| Ackland, J. McKno ... ... ... ... ... ... | 1 Barnfield Crescent, Exeter. |
| *Ackland, W. R. ... ... ... ... ... ... ... | 5 Rodney Place, Clifton. |
| Adye, W. J. A. ... ... ... ... ... ... ... ... .. | Church House, Bradford, Wilts. |
| Aldous, George F.... ... ... ... ... ... ... | Charlton House, Mannamead, Plymouth. |
| Aldridge, Charles, M.D., C.M. Aberd.... ... | Plympton. |
| *Alexander, D.A., M.B., Ch.B. Vict.... ... ... | 30 Berkeley Square, Clifton. |
| Ambrose, J., M.B., C.M. Aberd. ... ... ... ... | 138 Fishponds Road, Eastville, Bristol. |
| Aveline, H. T. S. ... ... ... ... ... ... ... | Somerset Asylum, Taunton. |
| *Ballance, H. Stanley, M.D. Lond. ... ... | 4 Royal Terrace, Weston-super-Mare. |
| Barber, M. C. ... ... ... ... ... ... ... | Staple Hill, Bristol. |
| Baretti, T. G. L'Enardi ... ... ... ... ... | 49 Royal York Crescent, Clifton. |
| *Barker, G. H., M.B., B.Sc. Lond. ... ... ... | 124 Redland Road, Bristol |
| *Baron, Barclay J., M.B., C.M. Ed.... ... ... | 16 Whiteladies Road, Clifton. |
| Basset, Walter ... ... ... ... ... ... ... | 24 Stow Hill, Newport, Mon. |
| Batten, Rayner W., M.D. Lond. ... ... ... | 1 Brunswick Square, Gloucester. |
| Batten, W. Smith... ... ... ... ... ... ... | West Hill, Calne. |
| Bayer Co., The ... ... ... ... ... ... ... ... | 19 St. Dunstan's Hill, London, E.C. |
| *Beaver, R. A., M.D., Ch.B. Vict., M.B. Lond. | 15 The Avenue, Clifton. |
| *Beddoe, John, M.D. Ed., B.A. Lond., F.R.S. | The Chantry, Bradford-on-Avon. |

### LIST OF SUBSCRIBERS.

| | |
|---|---|
| BERNARD, C. | 2 Spencer Terrace, Fishponds. |
| *BERRY, F. C., M.D., B.Ch., B.A. Dub. | Burnham, Somerset. |
| BERRY, JAMES, M.B., B.S. Lond. | 21 Wimpole Street, London, W. |
| BIAMONTI, SEBASTIANO | 27 Via Balbi, Genoa, Italy. |
| *BLACKER, A. E. | 20 Victoria Square, Clifton. |
| *BLACKER, E., M.D. Durh. | 8 Meridian Road, Redland. |
| *BLAGG, ARTHUR F., M.D. Durh. | 28 Caledonia Place, Clifton. |
| BOARD, J. H. | Hospital, Stoke-on-Trent. |
| BODMAN, HERVEY, M.D. Lond. | 3 Whiteladies Road, Clifton. |
| *BOWKER, G. E., M.D. Edin. | 11 North Parade, Bath. |
| BOYCE, JAMES G. | The Chipping, Wotton-under-Edge. |
| BOYD, GEO. S. J. | 19 St. George's Square, Stamford, Lincs. |
| *BRASHER, CHARLES W. J. | 128 Cotham Brow, Bristol. |
| *BRISTOWE, H. C., M.D. Lond. | Wrington, Somerset. |
| *BROOM, H., M.B., C.M. Glas. | 47 City Road, Bristol. |
| BROWN, G. ARTHUR | Tredegar. |
| BROWN, WILLIAM, M.D., C.M. Glasg. | Park View, Fishponds, Gloucestershire. |
| *BULLEN, F. ST. JOHN | 12 Pembroke Road, Clifton. |
| *BUNBURY, E. G. | 13 Dowry Square, Hotwells. |
| *BURD, E. LYCETT, M.D., B.C. Cantab. | Shirehampton. |
| BURROUGHS, E. F. H. | 3 Elgin Park, Bristol. |
| *BUSH, J. PAUL, C.M.G. | Vyvyan House, Clifton Park, Clifton. |

| | |
|---|---|
| *CAMPBELL-HORSFALL, C.E., M.B., Ch.B. (Vict.) | Rossmore, Cheltenham. |
| *CARTER, T. M. | 28 Victoria Square, Clifton. |
| *CARWARDINE, THOMAS, M.B., M.S. Lond. | 16 Victoria Square, Clifton. |
| *CAVE, EDWARD J., M.D. Lond. | 20 Circus, Bath. |
| *CHARLES, J. R., M.A., M.D., B.C. Cantab. | 12 Victoria Square, Clifton. |
| CHUTE, HENRY M. | King William's Town, S. Africa. |
| *CLARK, W. RONALDSON, M.A., M.B., B.C. Aberd. | Belvedere, Weston-super-Mare. |
| *CLARKE, JOHN MICHELL, M.D., M.A. Cantab. | 28 Pembroke Road, Clifton. |
| COBBLEDICK, A. S., M.D., B.S. Lond. | 402 Brixton Road, London, S.W. |
| COCHRAN, ALEXANDER, M.B., C.M. Glasg. | Bath Road, New Brislington, Bristol. |
| *COLERIDGE, ALFRED, M.B., B.S. Lond. | 48 Coronation Road, Bristol. |
| *COOK, HENRI, M.D., C.M. Aberd. | 1 Dean Lane, Southville, Bristol. |
| *COOMBS, CAREY, M.D. Lond. | 74 St. Paul's Road, Clifton. |
| *CORRIGAN, W. J. | Cloughmore, Splott Avenue, Cardiff. |
| COSSHAM, W. R., M.D., C.M. Aberd. | Cirencester. |
| COTTON, WILLIAM, M.B., C.M., M.A. Ed. | 231 Gloucester Road, Bristol. |
| COULSON, THOS. E., M.B., C.M. Edin. | |
| COULTER, R. J., M.B. Dub. | 11 Clytha Park Road, Newport, Mon. |
| COUSINS, J. WARD, M.D. Lond. | Kent Road, Southsea. |
| COWAN, F. S. | 27 Queen Square, Bath. |
| CRIDLAND, A. B. | 52 Waterloo Road, Wolverhampton. |
| *CROSS, F. RICHARDSON, M.B. Lond. | Worcester House, Clifton. |
| *CROSSMAN, F. W. | Hambrook, Gloucestershire. |
| *CROUCH, C. PERCIVAL, M.B. Lond. | 1 Royal Terrace, Weston-super-Mare. |
| CULROSS, JAMES, M.B., C.M., M.A. Glasg. | 66 Queen Street, Newton Abbot. |

| | |
|---|---|
| *DACRE, JOHN | 14 Eaton Crescent, Clifton. |
| *DAVIES, D. S., M.D. Lond., D.P.H. Cantab. | 60 Oakfield Road, Clifton. |
| DENING, EDWIN | Manor House, Stow-on-the-Wold. |

DEVINE, H. ... ... ... ... ... ... ... ... London County Asylum, Cane Hill, Coulsdon, Surrey.
DICKIE, JAMES STEEL, M.D., C.M. Aberd. ... Boscombe Park, Bournemouth.
*DOBSON, NELSON C. ... ... ... ... ... ... ... Avonmore, Sneyd Park.
DUNBAR, ELIZA L. WALKER, M.D. Zürich ... 9 Oakfield Road, Clifton.
*DUNKLEY, E. V., M.B., B.S. Lond. ... ... ... 7 Buckingham Place, Clifton.
DYMOKE, F. ... ... ... ... ... ... ... ... ... 21 Cotham Road, Bristol.

EADON, W. F. BAILEY ... ... ... ... ... ... ... Hambrook, Gloucestershire.
*EAGER, REGINALD, M.D. Lond. ... ... ... ... Northwoods, Winterbourne.
EBERLE, EMILY, M.A., M.B., B.Ch. Dub. ... 17 Oakfield Road, Clifton.
*EDGEWORTH, F. H., M.B., B.C., B.A. Cantab., D.Sc. Lond. ... ... ... ... ... ... ... ... 35 Pembroke Road, Clifton.
EDWARDS, E. S. ... ... ... ... ... ... ... ... Empingham, Stamford.
ELDER, WILLIAM ... ... ... ... ... ... ... ... 4 Trelawney Road, Bristol.
ELLIOT BROS. LTD. ... ... ... ... ... ... ... *Australian Pharmaceutical Notes & News.* O'Connell Street, Sydney, N.S.W.
*ELLIOTT, CHRISTOPHER, M.D., B.A. Dub. ... 3 Beaufort Road, Clifton.
ELLIOTT, F. PERCY, M.B., C.M. Aberd. ... ... 113 Grove Road, Walthamstow, London. N.E.
*ELLIOTT, W. H. A., M.B., B.S. Lond. ... ... Bristol Dispensary, Castle Green.
EVANS, T. G. C. ... ... ... ... ... ... ... ... Budleigh Salterton, Devon.
*EVERY-CLAYTON, L. E. V., M.D. Lond. ... ... Rosslyn, Clevedon, Somerset.

*FALCONER, A. W., M.D. Aberd. ... ... ... Bristol Royal Infirmary.
FAIRBANKS, W., M.D., C.M. Ed. ... ... ... ... Wells, Somerset.
*FELLS, A., M.B., C.M. Edin. ... ... ... ... 107 Ashley Road, Bristol.
*FERGUSON, R. S., B.A. Durh., M.B. C.M. Edin. Elm Grove, Calne, Wilts.
*FIRTH, J. L., M.D., M.S. Lond. ... ... ... ... 8 Victoria Square, Clifton.
FISHER, THEODORE, M.D. Lond. ... ... ... North Eastern Hospital, London.
*FLECK, D., M.B., B.Ch., R.U.I. ... ... ... ... Brentry Inebriate Home, Westbury-on-Trym.
*FLEMMING, A. L. ... ... ... ... ... ... ... ... 34 Alma Road, Clifton.
*FLEMMING, C. E. S. ... ... ... ... ... ... ... Manvers House, Bradford-on-Avon.
*FLETCHER, J., M.D., C.M., D.P.H. Aberd. ... Ham Green, Somerset.
FLOOD, P. ... ... ... ... ... ... ... ... ... ... 318 Stapleton Road, Bristol.
*FORTESCUE-BRICKDALE, J. M., M.A., M.D., B.Ch. Oxon. ... ... ... ... ... ... ... ... 52 Pembroke Road, Clifton.
FORTY, D. H. ... ... ... ... ... ... ... ... ... Wotton-under-Edge.
FOSS, E. V. ... ... ... ... ... ... ... ... ... Clouds Hill House, St. George, Bristol.
FOX, F. F. ... ... ... ... ... ... ... ... ... ... Yate House, Yate.
*FRASER, FORBES ... ... ... ... ... ... ... ... 2 The Circus, Bath.
FREEMAN, J., M.D. Brux. ... ... ... ... ... ... Glencairn Villa, Queen's Road, Clifton.

GARLAND, E. B., M.B., C.M. Edin. ... ... ... 114*a* Hampton Road, Redland.
GENGE, T. T. ... ... ... ... ... ... ... ... ... 21 Whiteladies Road, Bristol.
*GERRISH, D. S. ... ... ... ... ... ... ... ... 322 Church Road, St. George, Bristol.
*GIBBS, A. N. GODBY ... ... ... ... ... ... ... 52 Whiteladies Road, Clifton.

### LIST OF SUBSCRIBERS.

Goss, T. Biddulph... ... ... ... ... ... ... ... 1 The Circus, Bath.
*Goulden, C. B., M.A., M.B., M.C. Cantab. ... 9 Richmond Park Road, Clifton.
*Gratte, C. Brooke ... ... ... ... ... ... ... 19 Gold Tops, Newport, Mon.
*Green, T. A., M.D., C.M. Edin. ... ... ... 33*a* Whiteladies Road, Clifton.
*Greer, W. J. ... ... ... ... ... ... ... ... ... 19 Gold Tops, Newport, Mon.
*Grey, Harry, M.D., C.M.Edin. ... ... ... 492 Fishponds Road, Bristol.
Griffiths, Cornelius A. ... ... ... ... ... ... 6 Windsor Place, Cardiff.
*Griffiths, J. S. ... ... ... ... ... ... ... ... 20 Redland Park, Bristol.
*Griffiths, L. M. ... ... ... ... ... ... ... ... 11 Pembroke Road, Clifton.
*Groves, E. W. H., M.B., B.Sc. Lond. ... ... 16 Richmond Hill, Clifton.

Hadwen, W. R., M.D. St. And. ... ... ... ... 35 Brunswick Square, Gloucester.
*Hailes, Clements D. G., M.D., C.M. Ed. ... 27 Alma Road, Clifton.
*Haines, A. H. ... ... ... ... ... ... ... ... ... 12D Cotham Road, Bristol.
*Hall, E. G., M.B. Lond. ... ... ... ... ... 53 Hampton Road, Redland, Bristol.
*Hall, I. Walker, M.D., Ch.B.Vict. ... ... 9 Royal Park, Clifton.
Handfield-Jones, Montagu, M.D. Lond. ... 35 Cavendish Square, London, W.
*Harris, H. Elwin, B.A., M.B. Cantab. ... ... 13 Lansdown Place, Clifton.
*Harrison, A. J., M.B. Lond. ... ... ... ... Guthrie Road, Clifton.
*Harsant, W. H. ... ... ... ... ... ... ... ... Tower House, Clifton.
Hartnell, E. B. ... ... ... ... ... ... ... ... Thornbury, R.S.O., Glos.
*Harvey, Alfred, M.B. Lond. ... ... ... ... Westbury-on-Trym.
Hayman, Alfred G. ... ... ... ... ... ... ... Hapsford House, near Frome.
*Hayman, C. A., M.D. St. And. ... ... ... ... Kingston Villa, Richmond Hill, Clifton
Heaven, John C., D.P.H. Lond. ... ... ... 17 Whiteladies Road, Bristol.
*Herapath, C. K. C. ... ... ... ... ... ... ... Cotham Park, Bristol.
Hill, Hedley ... ... ... ... ... ... ... ... ... 1 Whiteladies Road, Clifton.
*Hill, Walter J. ... ... ... ... ... ... ... ... The Brow, Clevedon.
Hodges, W. ... ... ... ... ... ... ... ... ... ... 38 Park Road, Gloucester.
Holmes, Thomas E., M.A., M.B., B.C. Cantab. Rosendal, Redland Road, Bristol.
Hospital, Bristol General (*Sec.*, W. Thwaites).
Hospital, Taunton and Somerset (*House-Surgeon*, W. Drake).
Huggard, W. R., M.D., M.Ch., M.A. Roy. Univ. ... ... ... ... ... ... ... ... ... ... Davos-Platz, Switzerland.

Imlay, G. A., M.B., C.M. Aberd. ... ... ... 1 Cotham Park, Bristol.
Infirmary, Bristol Royal (*Secretary*, W. E. Budgett).
Ingle, C. Durban ... ... ... ... ... ... ... ... Somerton, Somerset.

Jamison, A., M.B., B.Ch., M.A. R.U.I. ... ... Woodstock Road, Belfast.
*Jeans, Fleet-Surgeon Austen, R.N. ... ... H.M.S. *Dædalus*, Bristol.
Jones, Evan ... ... ... ... ... ... ... ... ... Ty-mawr, Aberdare.
*Jones, E. J. Trevor, M.D. Brux. ... ... ... Aberdare, Glamorganshire.
*Jones, J. Ellington ... ... ... ... ... ... ... 16 Richmond Park Road, Clifton.
Jones, H. Macnaughton, M.D., M.Ch. R.U.I. 131 Harley Street, London, W.
*Jones, R. Llewellyn, M.B. Lond. ... ... ... 40 Gay Street, Bath.
Joseph, A. Hill, M.D. Lond. ... ... ... ... Bexhill-on-Sea.

*Keir, J. C., M.B., Ch.B. Ed. ... ... ... ... The Limes, Melksham.
*Kemm, F. St. J.... ... ... ... ... ... ... ... ... Worle.
*Kyle, H. G., M.B., B.Ch. Oxon. ... ... ... 31 Westbury Road, Westbury-on-Trym.

*Lace, F. ... ... ... ... ... ... ... ... ... ... 1 Paragon, Bath.
Lancaster, E. Le Cronier, B.A., M.B., B.Ch. Oxon. ... ... ... .. ... ... ... ... ... Winchester House, Swansea.
*Lansdown, R. G. P., M.B., B.S. Dur. ... ... 39 Oakfield Road, Clifton.
*Lavington, C. C., M.B., B.S. Dur. ... .. 1 Burlington Road, Redland.
Lawrie, J. Macpherson, M.D., C.M. Glasg.... Greenhill, Weymouth.
Le Soudier, H. ... ... ... ... ... ... ... ... 174 et 176 Boulevard St. Germain, Paris.
*Lees, E. Leonard, M.D., C.M. Ed. ... ... ... 1 Chesterfield Place, Clifton.
Leigh, W. W. ... ... ... ... ... ... ... ... ... Treharris, Glamorganshire.
*Leonard, R. C., M.D. Ed. ... ... ... ... ... 134 Coronation Road, Bristol.
Lowe, T. Pagan ... ... ... ... ... ... ... ... 16 Circus, Bath.
*Lucas, J. J. S., M.D. Lond. ... ... ... ... ... 186 Wells Road, Totterdown.
Lucy, Reginald H. ... ... ... ... ... ... ... 9 The Crescent, Plymouth.

*McCrea, P. W., M.D., B.Ch., B.A.O., B.A. Dub. ... ... ... ... ... ... ... ... ... ... 19 Portland Square, Bristol.
MacDonald, P. W., M.D., C.M. Aberd. ... ... Herrison, Dorchester.
Mackinnon, Charles, M.B., C.M., M.A. Glasg. Davaar, Cirencester.
Maclean, Ewen J., M.D., C.M. Ed.... ... ... 12 Park Place, Cardiff.
Maclean, J. Campbell, M.B., C.M. Ed.... ... Swindon House, Swindon, Wiltshire.
Macready, J. ... ... ... ... ... ... ... ... ... 42 Devonshire Street, Portland Place, London, W.
Marsh, O. E. Bulwer ... ... ... ... ... ... ... Parkdale, Newport, Mon.
Marshall, Arthur L., M.B., B.A. Cantab ... 206 Upper Clapton Road, London, N.E.
*Martin, E. F., M.B. Ed. ... ... ... ... ... ... 7 Royal Terrace, Weston-super-Mare.
*Martin, R C. ... ... ... ... ... ... ... ... ... 3 Clifton Vale, Clifton.
*Martyn, G. J. King, M.D., B.C., B.A. Cantab. ... ... ... ... ... ... ... ... ... ... 12 Gay Street, Bath.
Mason, S. B. ... ... ... ... ... ... ... ... ... 12 Park Terrace, Pontypool.
Mason, William ... ... ... ... ... ... ... ... Sedgemoor Cottage, St. Austell, Cornwall.
Matthews, Charles E., M.D. Dur. ... ... St. Aubyn's Park, Tiverton.
Meredith, John, M.D. Ed. ... ... ... ... ... ... Wellington, Somerset.
*Mole, Harold F. ... ... ... ... ... ... ... ... 19 Mortimer Road, Clifton, Bristol.
Monckton, William ... ... ... ... ... ... ... Portishead.
*Moore, L. A. ... ... ... ... ... ... ... ... ... 45 Ashley Road, Bristol.
Moores, De la Hey ... ... ... ... ... .. ... 6 West Mall, Clifton.
Morgan, Albert T., M.D. Brux. ... ... ... 89 Chesterfield Road, St. Andrew's Park, Bristol.

| | |
|---|---|
| *MORGAN, T. WHITWORTH S. ... ... ... ... | The Laurels, Timsbury, Bath. |
| *MORTON, C. A. ... ... ... ... ... ... ... ... ... | 14 Vyvyan Terrace, Clifton. |
| *MORTON, W. B., M.D. Lond. ... ... ... ... | Brislington House, Brislington. |
| MUNDEN, CHARLES ... ... ... ... ... ... ... | Ilminster. |
| *MUTHU, CHOWRY, M.D. Brux. ... ... ... ... | Mendip Hills Sanatorium, Wells. |
| *MYLES, G T. ... ... ... ... ... ... ... ... | 147 Whiteladies Road, Bristol. |
| *NEILD, NEWMAN, M.B., B.Ch. Vict. ... ... ... | 9 Richmond Hill, Clifton. |
| NEVILL, W. N., M.D., B.Ch., B.A. Dub. ... ... | 12 Acraman's Road, Southville, Bristol. |
| *NEWMAN, CHARLES ... ... ... ... ... ... ... | 156 Cheltenham Road, Bristol. |
| *NEWMAN, HERBERT, M.B., B.S.Dur. ... ... | 102 Coronation Road, Bristol. |
| *NEWNHAM, W. H. C., M.B., M.A. Cantab. ... | 3 Lansdown Place, Victoria Square, Clifton. |
| NICHOLSON, T. D., M.D. Ed. ... ... ... ... | 2 Whiteladies Road, Clifton. |
| *NIXON, J. A., B.A., M.B., B.C. Cantab. ... ... | 18 West Mall, Clifton. |
| NORMAN, J. H. ... ... ... ... ... ... ... ... | Goodleigh, Winkleigh, Devonshire. |
| ODELL, WILLIAM, M.D. Durh. ... ... ... ... | Ferndale, Torquay. |
| *OGILVY, ALEXANDER, M.D., B.Ch., B.A. Dub. | Leny, Clifton Park, Clifton. |
| OPENSHAW, E. H. ... ... ... ... ... ... ... | Cheddar. |
| *ORMEROD, H. LAWRENCE, M.B., B.Ch. R.U.I. | 2 Henleaze Road, Westbury Park. |
| PAGE, G. SHEPLEY ... ... ... ... ... ... ... ... | 78 Old Market Street, Bristol. |
| PAPILLON, J. W. ... ... ... ... ... ... ... ... | Brent Knoll, Somerset. |
| *PARKER, GEORGE, M.D., M.A. Cantab. ... ... | 14 Pembroke Road, Clifton. |
| *PARSONS, H. F.... ... ... ... ... ... ... ... | The Heath, Stoke Bishop. |
| PARSONS, J. H., M.B., B.S., D.Sc. Lond.... ... | 27 Wimpole Street, London, W. |
| PATERSON, DONALD ROSE, M.D., C.M. Ed. ... | 18 Windsor Place, Cardiff. |
| PEAKE, ARTHUR W.... ... ... ... ... ... ... | 34 Gloucester Road, Bishopston, Bristol. |
| PEAKE, G. ARTHUR ... ... ... ... ... ... ... | Rodney Place, Cheltenham. |
| PEARSE, E. M. ... ... ... ... ... ... ... ... | 2 Varley Terrace, Liskeard. |
| PECK, Lt.-Col. F.S., I.M.S. ... ... ... ... | 6 Harington Street, Calcutta. |
| PERDRAU, J. A., M.B. Lond. ... ... ... ... | 99 Gower Street, London, W.C. |
| PHILLIPS, C. M., M.D. Brux. ... ... ... ... | Ravenslea, Kensington Hill, Brislington. |
| PHILLIPS, EDWARD PICTON ... ... ... ... ... | High Street, Haverfordwest. |
| *PICKERING, C. F. ... ... ... ... ... ... ... | 13 Berkeley Square, Bristol. |
| POWELL, J. J., M.D. Lond. ... ... ... ... ... | 2 Gloucester Road, Bishopston, Bristol |
| *POWELL, L. W. ... ... ... ... ... ... ... ... | Two Mile Hill Road, Kingswood, Bristol |
| POWELL, WILLIAM, M.B. Lond. ... ... ... ... | Hill Garden, Torquay. |
| PRICE, D. T., M.B. Lond. ... ... ... ... ... | Castle Cary, Somerset. |

### LIST OF SUBSCRIBERS.

*Prichard, Arthur W. ... ... ... ... ... ..... 6 Rodney Place, Clifton.
*Prowse, Arthur B., M.D. Lond. ... ... ... 5 Lansdown Place, Clifton.
Prowse, W. B.... ... ... ... ... ... ... ... 11 St. George's Place, Brighton.

Randolph, Charles ... ... ... ... ... ... St. Michael's, Milverton, Somerset.
*Rattray, J. M., M.D., C.M., M.A. Aberd. ... Frome.
*Rayner, D. C. ... ... ... ... ... ... ... ... ... 9 Lansdown Place, Clifton, Bristol.
Redwood, T. Hall, M.D., C.M., M.A.Dur. ... The Lawn, Rhymney, Monmouthshire.
Rendall, John ... ... ... ... ... ... ... Forestside, Lymington.
Richards, D. T. ... ... ... ... ... ... ... The Meadows, Risca, Mon.
*Ridge, Percy B., M.B., B.Ch., R.U.I. ... ... Children's Hospital, Bristol.
Ritchie, Thomas P. ... ... ... ... ... ... 3 Redcliff Parade West, Bristol.
*Rogers, B. M. H., M.D., B.Ch., B.A. Oxon. ... 1 Victoria Square, Clifton.
*Rolfe, R., B.A., M.B., B.C. Camb. ... ... ... Woodhall House, Avonmouth.
*Rose, F. H. ... ... ... ... ... ... ... ... 13 Westfield Park, Clifton.
*Rossiter, George F., M.B. Lond. ... ... Cairo Lodge, Weston-super-Mare.
*Roué, W. Barrett, M.D., M.S. Dur. ... ... 2 Buckingham Place, Clifton.
*Roxburgh, Robert, M.B. Ed. ... ... ... 1 Victoria Buildings, Weston-super-Mare
*Rudge, C. King ... ... ... ... ... ... ... 145 Whiteladies Road, Bristol.
Ruffer, M. Armand, M.D., B.S., M.B. Oxon Ramleh, Alexandria, Egypt.
Rutherfoord, H. T., M.A., M.D. Camb. ... Salisbury House, Taunton.

Scott, Richard J. H. ... ... ... ... ... ... 28 Circus, Bath.

Seward, W. J., M.B. Lond. ... ... ... ... Colney Hatch Asylum, New Southgate, London, N.

*Shaw, J. E., M.B., C.M. Ed. ... ... ... ... 23 Caledonia Place, Clifton.

*Sheppard, A. L., M.B., B.S.Dur. ... ... ... Bristol Royal Infirmary.

*Sheppard, E. J. .. ... ... ... .. ... ... 46 St. Paul's Road, Clifton.

*Shoolbred, W. A. ... ... ... ... ... ... ... St. Ann's, Chepstow.

*Short, A. R., M.D., B.S., B.Sc. Lond. ... ... 21 Tyndall Avenue, Clifton.

*Sieveking, A. R. ... ... ... ... ... ... ... 18 Blenheim Road, Redland.

Skelton, Henry, M.D. St. And. ... ... ... Downend, Gloucestershire.

Smith, F. Shingleton, B.A., B.C.Cantab. ... Grindlay & Co., Bombay.

*Smith, G. Cockburn, M.D. Brux. ... ... ... 14 South Road, Newton Abbot.

*Smith, G. Munro ... ... ... ... ... ... ... 18 Apsley Road, Clifton.

*Smith, L. Shingleton ... ... ... ... ... ... Clifton Park, Clifton.

Smith, Rev. Reginald, M.A. Cantab. ... ... The Vicarage, Walpole St. Andrew, Norfolk.

*Smith, R. Shingleton, M.D., B.Sc. Lond. ... Clifton Park, Clifton.

Smith, W., M.B. Lond.... ... ... ... ... ... Brabourne, Ashford, Kent.

Smith, W. Alexander, M.B., M.A. Oxon. ... Newport, Essex.

Society, Cardiff Medical (*Hon. Lib.*, Queen Street, Cardiff).

### LIST OF SUBSCRIBERS.

| | |
|---|---|
| Society, Manchester Medical ... ... ... | Medical Society's Library, The Owens College, Manchester. |
| Society, Plymouth Medical (*Hon. Lib.*, J. Elliott Square). | |
| *Stack, E. H. E., B.A., M.B., B.C. Cantab. ... | Arvalee, Clifton Down Road, Clifton. |
| Staple, J. D. ... ... ... ... ... ... ... ... | Clevedon Villa, Cheltenham Road, Bristol. |
| Statham, R. Whiteside ... ... ... ... ... | Cheddar, Somersetshire. |
| *Steele, Charles, M.D. Dur. ... ... ... ... | 17 Richmond Hill, Clifton. |
| Stevens, W. H. ... ... ... ... ... ... ... ... | 26 Zetland Road, Bristol. |
| Stock, P. G. ... ... ... ... ... ... ... ... ... | Box 1049, Johannesburg, South Africa. |
| *Stock, W. S. V., M.B., B.S. Lond. ... ... ... | Montrose House, Queen's Road, Clifton. |
| Stoddart, F. Wallis ... ... ... ... ... ... ... | Southey House, College Green, Bristol. |
| *Swain, James, M.D., M.S. Lond. ... ... ... | 4 Victoria Square, Clifton. |
| *Swayne, W. Carless, M.D. Lond. ... ... ... | St. Paul's Road, Clifton. |
| *Symes, J. O., M.D. Lond. ... ... ... ... ... | 71 Pembroke Road, Clifton. |
| Symonds, H. P. ... ... ... ... ... ... ... ... | 35 Beaumont Street, Oxford. |
| Symons, John ... ... ... ... ... ... ... ... | Buriton House, Penzance. |

| | |
|---|---|
| Taylor, A. L. ... ... ... ... ... ... ... ... | 22 Downfield Road, Clifton. |
| Taylor, C. Bell, M.D. Edin. ... ... ... ... | 9 Park Row, Nottingham. |
| Taylor, C. J. ... ... ... ... ... ... ... ... | General Hospital, Hereford. |
| *Taylor, James ... ... ... ... ... ... ... ... | 36 Alma Road, Clifton. |
| *Temple, G. H., M.B., C.M. Ed. ... ... ... | Weston-super-Mare. |
| Thin, James ... ... ... ... ... ... ... ... ... | 54 & 55 South Bridge, Edinburgh. |
| Thomas, A. Garrod, M.D., C.M. Ed. ... ... | Clytha Park, Newport, Mon. |
| *Thomas, J. D., M.B. Cantab. ... ... ... ... | Northwoods, Winterborne, |
| Thomas, J. Lynn ... ... ... ... ... ... ... | 21 Windsor Place, Cardiff. |
| *Thomas, J. M. M. ... ... ... ... ... ... ... | 5 Colston Parade, Redcliffe, Bristol. |
| Thomson, Eustace B., M.D., C.M. Glasg. ... | Albany Place, Plymouth. |
| Tivy, W. J. ... ... ... ... ... ... ... ... ... | 5 Victoria Square, Clifton. |
| Tosswill, Louis H., M.B., B.A. Cantab.... ... | 34 West Southernhay, Exeter. |
| Tyley, R. P., M.D. St. And.... ... ... ... ... | Wedmore. |

| | |
|---|---|
| Vachell, Herbert R., M.D., C.M. Aberd. ... | 18 Newport Road, Cardiff. |
| *Vickery, W. H. ... ... ... ... ... ... ... | 1 Trewartha Park, Weston-super-Mare. |

| | |
|---|---|
| *Waldo, Henry, M.D., C.M. Aberd. ... ... ... | 19 Pembroke Road, Clifton. |
| *Walker, Cyril H., M.B., B.A. Cantab. ... ... | 8 Oakfield Road, Clifton. |
| Wallace, Rev. Canon, M.A. Oxon. ... ... ... | 3 Gloucester Row, Clifton. |
| *Wallace, John ... ... ... ... ... ... ... | Lauriston, 15 Knightstone Road, Weston-super-Mare. |
| Wallace, J. T., M.B., B.Ch., B.A. R.U.I. ... | 48 Coronation Road, Bedminster. |
| *Wallis, G. F. C., M.B,, Ch.B. Edin. ... ... | Bristol Eye Hospital. |
| *Walters, C. F. ... ... ... ... ... ... ... | 19 Whatley Road, Clifton. |
| Ward, J. L. W.... ... ... ... ... ... ... ... | Victoria Street, Merthyr Tydvil. |
| *Waterhouse, R., M.D. Lond. ... ... ... ... | 32 St, James's Square, Bath. |
| Webber, William W. ... ... ... ... ... ... | Crewkerne. |
| *Westcott, Staff-Surgeon W. G. ... ... ... | H.M.S. *Adventure*, Harwich. |
| *Whicher, A. H. ... ... ... ... ... ... ... | 115 Queen's Road, Clifton |

| | |
|---|---|
| White, C. R. ... ... ... ... ... ... ... ... | Nixon Villa, Merthyr Vale, Glamorgan. |
| White, J. W. ... ... ... ... ... ... ... ... | Orchard House, Nailsea. |
| *White, Percy W. ... ... ... ... ... ... ... | College Green, Bristol. |
| Wigan, C. A., M.D. Dur. ... ... ... ... ... | Portishead. |
| Wigmore, J., M.D. Roy. Univ. I. ... ... ... | 20 Green Park, Bath. |
| Wilcox, Henry, M.B. Aberd. ... ... ... ... | Fleet, Hampshire. |
| Willcox, R. Lewis ... ... ... ... ... ... | Warminster. |
| Willett, George G. D. ... ... ... ... ... ... | Keynsham, Somerset. |
| *Williams, E. C., M.B., B.C., B.A. Cantab. ... | 159 Whiteladies Road, Clifton. |
| Williams, H. Egerton ... ... ... ... ... | The Mount, Newport, Mon. |
| *Williams, P. Watson, M.D. Lond.... ... ... | 4 Clifton Park, Clifton. |
| *Williams, W. Roger ... ... ... ... ... ... | Beaufort House, Clifton Down, Clifton. |
| Willis, W. M.... ... ... ... ... ... ... ... ... | 7 Regent Street, Nottingham. |
| *Wills, W. K., M.B., B.C. Camb. ... ... ... | 19 Whiteladies Road, Clifton. |
| *Windsor-Aubrey, H. W. ... ... ... ... ... | Coronation Road, Bristol. |
| Woollcombe, Walter L. ... ... ... ... ... | 16 The Crescent, Plymouth. |
| *Wright, A. J., M.B., B.S. Lond. ... ... ... | 3 Codrington Place, Clifton. |

### *PUBLICATIONS RECEIVED.*

From Edward Arnold, London:

*The Chemical Basis of Pharmacology.* By Francis Francis and J. M. Fortescue-Brickdale M.A., M.D. 1908. Price, 14s. net.

From Baillière, Tindall & Cox, London:

*Aids to Surgery.* By Joseph Cunning. Second Edition. 1908. Price, 4s. net.
*The Pocket Anatomy.* Sixth Edition, revised and edited by C. H. Fagge, M.B., M.S. 1908. Price, 3s. 6d. net.
*Protozoa and Disease.* By J. Jackson Clarke, M.B. Part II. 1908. Price, 7s. 6d. net.

From John Bale, Sons & Danielsson, Ltd., London:

*Some of the Clinical Aspects of Pneumonia.* By Donald W. C. Hood, C.V.O., M.D. 1907.
*Stray Leaves, and some Fruit on Cancer and Tuberculosis.* By Henry D. McCulloch, M.B. 1907. Price, 10s. 6d. net.

From Cassell and Company, Limited, London:

*The Romance of Medicine.* By Ronald Cambpell Macfie, M.A., M.B, C.M. 1907 Price, 6s.

From J. & A. Churchill, London:

*Anatomical Terminology, with special reference to the* [B.N.A.]. By Lewellys F. Barker, M.D. 1907. Price, 5s. net.
*The Care of the Teeth in Public Elementary Schools.* By C. Edward Wallis, L.D.S. 1908 Price, 1s. net.

From Archibald Constable and Co., Ltd., London:

*The Integrative Action of the Nervous System.* By Charles S. Sherrington, D.Sc., M.D., F.R.S. 1906.

From the Joint Committee of Henry Frowde and Hodder and Stroughton, London:

*Rotunda Practical Midwifery.* By E. Hastings Tweedy, M.D., and G. T. Wrench, M.D. 1908. Price, 16s. net.
*Fevers in the Tropics.* By Leonard Rogers, M.D. 1908. Price, 30s. net.
*Life Insurance and General Practice.* By E. M. Brockbank, M.D. 1908. Price, 7s. 6d. net.

From William Heinemann, London:

*The Prolongation of Life.* By Élie Metchnikoff. The English translation edited by P. Chalmers Mitchell, M.A., D.Sc. 1907 Price, 12s. 6d. net.

From J. F. Lehmann, Munich:

*Drei Jahre Gallensteinchirurgie.* Von Prof. Dr. Hans Kehr, Dr. Liebold und Oberarzt. Dr. Neuling. 1908.

From H. K. Lewis, London:

*The Opsonic Method of Treatment.* By R. W. Allen, M B., B.S. 1907. Price, 5s. net.
*A Guide to the Administration of Ethyl-Chloride.* By G. A. H. Barton, M.D. Second Edition. 1907 Price, 2s.
*An Essay upon Disease: its Cause and Prevention.* By G. E. Richmond, M.D. 1907. Price, 2s. net.
*Studies in Blood-Pressure.* By George Oliver, M.D. Second Edition, enlarged. 1908. Price, 4s. net.
*Tumours of the Cerebellum.* By John Wyllie, M.D. 1908. Price, 4s. net.
*Diseases of the Nose and Throat.* By Herbert Tilley. 1908. Price, 14s. net.

From Longmans, Green and Co., London:

*Proceedings of the Royal Society of Medicine.* Vol. I., No. 1. November, 1907. Price, 7s. 6d. net.

From Masson et Cie, Paris:

*Traité du Paludisme.* Par A. Laveran. Deuxième Édition. 1907. Price, 12 fr.
*Les Venins. Les Animaux venimeux et la Sérothérapie antivenimeuse.* Par A. Calmette. 1907. Price, 12 fr.

From Young J. Pentland, Edinburgh:

*The Edinburgh Medical Journal.* New Series. Volume XXII. 1907.

From F. C. V. Vogel, Leipzig:

*Atlas der klinischen Mikroskopie des Blutes.* Zweite Auflage. Bearbeitet von Privatdozent Dr. E. Meyer und Professor Dr. H. Rieder. 1907. Price, M. 15.

From John Wright and Co., Bristol:

*First Aid.* By F. J. Warwick, B.A., M.B., and A. C. Tunstall, M.D. Fifth Edition. 1908. Price, 1s. net.
*The Experimental Prophylaxis of Syphilis.* By Dr. Paul Maisonneuve. Translated, and with an Introduction by Fernand L. de Verteuil, M.B. 1908. Price, 4s. net.

Bristol Medico-Chirurgical Journal,
MARCH, 1908.

### EXCHANGE-LIST.

### AFRICA.

### CAPE COLONY.

*Monthly.*

*South African Medical Record.* Cape Town: Townshend Taylor & Snashall.

### AMERICA.

### ARGENTINE REPUBLIC.

*Weekly.*

*La Semana Médica.* Buenos Ayres: 737 Callao.

### CANADA

*Monthly.*

*The Canada Lancet.* Toronto: The Ontario Publishing Co. Ltd.

*The Canadian Practitioner and Review.* Toronto: 61 Queen St. East.

*The Montreal Medical Journal.* Montreal: The Gazette Printing Company.

### MEXICO.

*Twice a month.*

*Gaceta Médica.* Mexico: Apartado postal, 517.

### PERU.

*Twice a month.*

*La Crónica Médica.* Lima: Imprenta Librería y Encuadernación de San Pedro.

### UNITED STATES.

*Weekly.*

*Boston Medical and Surgical Journal.* Boston: D. C. Heath & Co.

*Medical Record.* New York: William Wood & Co.

*The Lancet-Clinic.* Cincinnati: 319 West 7th Street

*Monthly.*

*American Medicine.* Philadelphia: 130 South Fifteenth Street.

*Archives of Pediatrics.* New York: E. B. Treat & Co.

*Buffalo Medical Journal.* Buffalo: 238 Delaware Avenue.

*Bulletin of the Johns Hopkins Hospital.* Baltimore: The Johns Hopkins Press.

*California State Journal of Medicine.* San Francisco 2210 Jackson Street.

*New York State Journal of Medicine.* New York: The Medical Society of the State of New York.

*The American Journal of Obstetrics.* New York: William Wood & Co.

*The American Journal of Ophthalmology.* St. Louis: 3036 Locust Street.

*The Monthly Cyclopædia of Practical Medicine.* Philadelphia: The F. A. Davis Co.

*The Post-Graduate.* New York: 2nd Avenue and 20th Street.

*The Therapeutic Gazette.* Detroit: E. G. Swift.

*Univ. of Penna. Medical Bulletin.* Philadelphia University of Pennsylvania Press.

*Quarterly.*

*The Alienist and Neurologist.* St. Louis: 3872 Washington Boulevard.

*Irregularly.*

*The Journal of Experimental Medicine.* New York The Macmillan Company.

*The Journal of Medical Research.* Boston: 240 Longwood Avenue.

*The Journal of Mental Pathology.* New York: State Press.

*Yearly.*

*Smithsonian Report.* Washington: Government Printing Office.

*Studies from the Department of Pathology of the College of Physicians and Surgeons—Columbia University* New York: 437 West 59th Street.

*Transactions of the American Association of Obstetricians and Gynecologists.* Philadelphia: William J. Dornan.

*Transactions of the American Dermatological Association.* New York: The Grafton Press.

*Transactions of the American Laryngological Association.* New York: Rooney & Otten Printing Co.

*Transactions of the American Ophthalmological Society.* Hartford: The Case, Lockwood & Brainard Co.

*Transactions of the American Pediatric Society* New York: A. G. Sherwood & Co.

*Transactions of the American Surgical Association* Philadelphia: William J. Dornan.

*Transactions of the Association of American Physicians.* Philadelphia: William J. Dornan.

*Transactions of the College of Physicians of Philadelphia.* Philadelphia: William J. Dornan.

### ASIA

### CHINA.

*Quarterly.*

*The China Medical Missionary Journal.* Shanghai 18 Peking Road.

### INDIA.

*Monthly.*

*The Antiseptic.* Madras: U. Rama Rau.

*The Indian Medical Gazette.* Calcutta: Thacker Spink & Co.

*Practical Medicine* Delhi: The Medico-Scientific Press.

### JAPAN.

*Monthly.*

*The Sei-I-Kwai Medical Journal.* Tokio Atago Chō Nichōme Shiba-Ku.

PHILIPPINE ISLANDS.

*Bi-monthly.*

*The Philippine Journal of Science.* Manila: Bureau of Printing.

### AUSTRALASIA.

AUSTRALIA.

*Monthly.*

*Intercolonial Medical Journal of Australasia.* Melbourne: Stillwell and Co.

*The Australasian Medical Gazette.* Sydney 121 Bathurst Street.

NEW ZEALAND.

*Quarterly.*

*New Zealand Medical Journal.* Wellington B.M.A. Wellington Branch).

### EUROPE

AUSTRIA.

*Weekly.*

*Wiener klinische Wochenschrift.* Vienna: Wilhelm Braumüller.

BELGIUM.

*Monthly.*

*Bulletin de l'Académie royale de Médecine de Belgique.* Brussels F. Hayez.

BRITISH ISLES.

*Weekly.*

*British Medical Journal.* London: 429 Strand.

*Medical Press and Circular.* London: A. A. Tindall.

*The Clinical Journal.* London: The Medical Publishing Company, Limited.

*The Hospital.* London: The Scientific Press (Limited).

*The Lancet.* London: 423 Strand.

*The Sanitary Record.* London: The Sanitary Publishing Co., Ltd.

*Fortnightly.*

*The Journal of Tropical Medicine.* London: John Bale, Sons & Danielsson, Ltd.

*Monthly.*

*Edinburgh Medical Journal.* Edinburgh: Young J. Pentland.

*Public Health.* London: The Incorporated Society of Medical Officers of Health.

*The Birmingham Medical Review.* Birmingham: Percival Jones, Ltd.

*The British Journal of Dermatology.* London: H. K. Lewis.

*The Glasgow Medical Journal.* Glasgow: Alex. Macdougall.

*The Journal of Laryngology, Rhinology, and Otology* London: Adlard & Son.

*The Medical Review.* London: 66 Finsbury Pavement.

*The Medical Chronicle.* Manchester Sherratt & Hughes.

*The Polyclinic.* London: John Bale, Sons & Danielsson, Ltd.

*The Practitioner.* London: 149 Strand.

*The Proceedings of the Royal Society of Medicine* London: Longmans, Green & Co.

*The Scottish Medical and Surgical Journal.* Edinburgh: William Green & Sons.

*Quarterly.*

*Journal of Mental Science.* London: J. & A. Churchill.

*The British Journal of Tuberculosis.* London: Baillière, Tindall & Cox.

*The Caledonian Medical Journal.* Glasgow: Alex. Macdougall.

*The Northumberland and Durham Medical Journal.* Newcastle-upon-Tyne: The "Daily Journal" Office.

*The West London Medical Journal.* London: John Bale, Sons & Danielsson, Ltd.

*Half-yearly.*

*The Liverpool Medico-Chirurgical Journal.* Liverpool: Medical Institution.

*Yearly.*

*Guy's Hospital Reports.* London: J. & A. Churchill

*King's College Hospital Reports.* London: Adlard & Son.

*Ophthalmological Transactions.* London: J. & A. Churchill.

*St. Bartholomew's Hospital Reports.* London: Smith, Elder & Co.

*St. Thomas's Hospital Reports.* London: J. & A. Churchill.

*The Medical Annual.* Bristol: John Wright & Co.

*The Medical Society's Transactions.* London: Harrison & Sons.

*The Transactions of the Edinburgh Obstetrical Society.* Edinburgh: Oliver and Boyd.

*The Westminster Hospital Reports.* London: Henry J. Glaisher.

*Transactions of the Royal Academy of Medicine in Ireland.* Dublin: John Falconer.

*Year-Book of Scientific and Learned Societies* London: C. Griffin & Co., Ltd.

DENMARK.

*Weekly.*

*Hospitalstidende.* Copenhagen: Jacob Lund.

FRANCE.

*Three times a week.*

*Gazette des Hôpitaux.* Paris: 49 Rue St.-André-des-Arts.

*Weekly.*

*Bulletin de l'Académie de Médecine.* Paris: Masson et Cie.

*Gazette des Hôpitaux de Toulouse.* Toulouse: 3 Avenue Lafayette.

FRANCE—*Weekly* (continued).

*Gazette hebdomadaire des Sciences médicales de Bordeaux.* Bordeaux: 8 Rue Paul-Bert.

*L'Écho médical du Nord.* Lille: 128 Boulevard de la Liberté.

*Le Progrès médical.* Paris: 14 Rue des Carmes.

*Revue hebdomadaire de Laryngologie, d'Otologie et de Rhinologie.* Bordeaux: G. Gounouilhou.

*Fortnightly.*

*Le Nord médical.* Lille: rue Solferino, 238.

*Twice a month.*

*Gazette de Gynécologie.* Paris: 8bis Rue de Châteaudun.

*Revue de Thérapeutique médico-chirurgicale.* Paris: Félix Alcan.

*Monthly.*

*Archives de Médecine et de Chirurgie spéciales.* Paris: Avenue Friedland, 22.

*Archives de Neurologie.* Paris: 14 Rue des Carmes.

*Journal d'Hygiène.* Paris: 79 Avenue de Wagram.

*Revue générale d'Ophtalmologie.* Paris: Masson et Cie.

*Revue pratique d'Obstétrique et de Gynécologie.* Paris: 31 Rue Pierre-Charron.

GERMANY.

*Weekly.*

*Zentralblatt für Chirurgie.* Leipzig: J. A. Barth.

*Zentralblatt für Gynäkologie.* Leipzig: J. A. Barth.

*Zentralblatt für innere Medicin.* Leipzig: J. A. Barth.

*Münchener medicinische Wochenschrift.* Munich: J. F. Lehmann.

GREECE.

*Monthly.*

*Ἰατρικὴ πρόοδος.* Syra: Renieri Brindesi.

*La Grèce médicale.* Syra: Renieri Brindesi.

*Three times a year.*

*Δελτίον τῆς ἐν Ἀθήναις Ἰατρικῆς Ἑταιρείας.* Athens: 85 rue de l' Université.

HOLLAND.

*Weekly.*

*Nederlandsch Tijdschrift voor Geneeskunde.* Amsterdam: F. van Rossen.

ITALY.

*Twice a month.*

*Giornale Internazionale delle Scienze Mediche.* Naples Enrico Detken.

*Bi-monthly.*

*Archivio di Ortopedia.* Milan: Via San Calimero, 31.

NORWAY.

*Monthly.*

*Norsk Magazin for Lægevidenskaben.* Christiania Steen'ske Bogtrykkeri.

PORTUGAL.

*Weekly.*

*A Medicina Contemporanea.* Lisbon: José Antonio Rodrigues.

RUSSIA.

*Weekly.*

*Medycyna.* Warsaw: Nowo-Jasna, Nr. 6.

*St. Petersburger medicinische Wochenschrift.* St. Petersburg: Carl Ricker.

*Monthly.*

*Finska Läkaresällskapets Handlingar.* Helsingfors: Helsingfors Centraltryckeri.

SPAIN.

*Weekly.*

*El Siglo Médico.* Madrid: Magdalena, 36.

SWEDEN.

*Bi-monthly.*

*Nordiskt Medicinskt Arkiv.* Stockholm: P. A. Norstedt & Söner.

TURKEY.

*Monthly.*

*Gazette médicale d'Orient.* Constantinople: "Levant Herald."

